# External evaluation of the prediction equation for milk fat yield by the 2021 NASEM dairy model using data from eastern Canadian dairy herds

**DOI:** 10.3168/jdsc.2022-0360

**Published:** 2023-07-13

**Authors:** S. Binggeli, D. Pellerin, P.Y. Chouinard

**Affiliations:** Département des sciences animales, Université Laval, Québec, QC, Canada G1V 0A6

## Abstract

In 2021, the National Academies of Sciences, Engineering, and Medicine (NASEM) issued an equation to predict milk fat yield using dairy cow characteristics and diet composition as input variables. This model was evaluated externally using a data set composed of 541 feed and production records obtained from 23 eastern Canadian dairy herds. The use of the developed equation requires the prediction of dry matter intake. Cow intake used in the model assessment has been obtained by NASEM equations based on (1) animal factors, or (2) a combination of feed composition and animal factors. The prediction of milk fat yield was shown to be accurate. The best prediction was obtained using intake estimated based solely on animal factors (concordance correlation coefficient = 0.68).

## Highlights

• The 2021 NASEM proposed an equation to predict milk fat yield.
• The model uses dairy cow characteristics and diet composition as input variables.
• An external evaluation has been conducted using records from Canadian dairy herds.
• The prediction of milk fat yield was shown to be precise and accurate.
• The model can be targeted for on-farm application.

## Graphical Abstract

**Figure:**
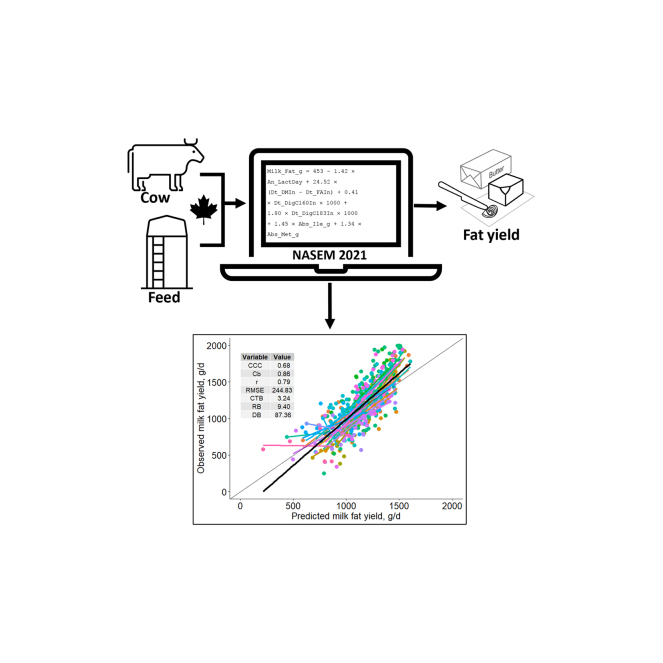
**Summary:** This communication presents the results of an evaluation of the 2021 National Academies of Sciences, Engineering, and Medicine (NASEM) model developed to predict milk fat yield using data from Canadian dairy operations. The model was shown to be accurate and therefore appears to be a valuable tool for estimating the impact of changes in diet composition on milk fat yield in commercial herds.

In Canada, milk yield is restricted by supply management based on butterfat production. According to the current milk payment scheme, fat is the most important economic component of milk, representing 58% of the price paid to producers in the province of Québec (Hounhouiga, 2022). It is therefore of primary importance to be able to predict the production of milk fat on commercial dairy farms. In the eighth revised edition of Nutrient Requirements of Dairy Cattle, the National Academies of Sciences, Engineering, and Medicine (**NASEM**) proposed an equation to predict milk fat yield (**MFY**) using animal characteristics and diet composition as input variables (NASEM, 2021). This multivariate equation was developed and cross-validated by Daley and Hanigan (2019) and Daley et al. (2022) and reads as follows:

*Milk_Fat_g* = 453 − (1.42 × *An_LactDay*) + [24.52 × (*Dt_DMIn* − *Dt_FAIn*)] + (0.41 × *Dt_DigC160In* × 1,000) + (1.80 × *Dt_DigC183In* × 1,000) + (1.45 × *Abs_Ile_g*) + (1.34 × *Abs_Met_g*),

where *Milk_Fat_g* = MFY (g/d), *An_LactDay* = DIM (d), *Dt_DMIn* = DMI (kg/d), *Dt_FAIn* = FA intake (kg/d), *Dt_DigC160In* = predicted digested 16:0 intake (kg/d), *Dt_DigC183In* = predicted digested 18:3 intake (kg/d), *Abs_Ile_g* = absorbed Ile (g/d), and *Abs_Met_g* = absorbed Met (g/d).

The objective of the present communication was to evaluate this model using an independent data set. We hypothesized that the NASEM (2021) model would accurately predict MFY using records from eastern Canadian dairy herds. Apart from feed composition, the use of the issued equation requires the determination or prediction of DMI of lactating cows. In this regard, the NASEM (2021) model proposes a first equation developed by de Souza et al. (2019) based solely on animal characteristics as follows:

[1] *Dt_DMIn_Lact1* = (3.7 + [5.7 × (*An_Parity* − 1)] + (0.305 × *Milk_NEuse_Target_*) + (0.022 × *AnBW*) + {−0.689 − [1.87 × (*An_Parity* − 1)]} × *AnBCS*) × [1 − ({0.212 + [0.136 × (*An_Parity* − 1)]} × e^(–0.053×^*^An_DayLact^*^)^)],

where *Dt_DMIn_Lact1* = DMI (kg/d), *An_Parity* = parity (1 = primiparous and 2 = multiparous), *Milk_NEuse_Target_* = net energy output for the desired milk yield (Mcal/d), *AnBW* = BW (kg), *AnBCS* = BCS (from 1 = thin to 5 = obese), and *An_DayLact* = DIM (d).

A second equation to predict DMI developed by Allen et al. (2019) is based on feed composition and animal factors, and reads as follows:

[2] *Dt_DMIn_Lact2* = 12.0 − (0.107 × *Dt_fNDF*) + (8.17 × *Dt_ADF/Dt_NDF*) + (0.0253 × *ForNDF48_NDF*) − {0.328 × [(*Dt_ADF*/*Dt_NDF*) − 0.602] × (*ForNDF48_NDF* − 48.3)} + (0.225 × *Milk_Prod_Target_*) + [0.00390 × (*ForNDF48_NDF* − 48.3) × (*Milk_Prod_Target_* − 33.1)],

where *Dt_DMIn_Lact2* = DMI (kg/d), *Dt_fNDF* = forage NDF content of the diet (%), *Dt_ADF* = ADF content of the diet (%), *Dt_NDF* = NDF content of the diet (%), *ForNDF48_NDF* = forage NDF digestibility (48-h in vitro incubation), and *Milk_Prod_Target_* = desired milk yield (kg/d). These 2 equations were compared in the current evaluation of MFY prediction.

All experimental procedures of this study were approved by the Animal Care Committee from Université Laval following the guidelines of the Canadian Council on Animal Care (2009). Data used for this analysis were collected from 23 commercial settings in the province of Québec, Canada, as previously described by Fadul-Pacheco et al. (2017) and Binggeli et al. (2022). Briefly, the visit of each farm was scheduled to occur simultaneously with a routine DHI test, from October 2015 to June 2016. Body weight was estimated from the thoracic circumference (Yan et al., 2009). Body condition score was not evaluated and was set to 2.5 for all cows. Milk yield was determined, and samples were collected using on-farm milk meters. Samples were stored at 4°C with bronopol as a preservative. Milk fat, protein, and lactose concentrations were determined by infrared spectroscopy (Foss MilkoScan FT 6000) by the Canadian DHI (Lactanet). Net milk energy output was calculated from milk composition using the NASEM (2021) equation. Ingredient compositions of diets were recorded, and feed samples were collected. Chemical composition of concentrates was determined by wet chemistry in a commercial laboratory (SGS Agrifood Laboratories). The forages were analyzed by infrared spectroscopy (Foss XDS Rapid Content Analyzer) by Lactanet. Forage NDF digestibility was estimated from this composition using the NASEM (2021) equation. For other missing nutrients (i.e., fatty acids and AA), table values from NASEM (2021) were employed.

A total of 541 feed and production records, all from a single farm visit, were retained for the analysis. Production target was set as observed milk yield. The model performances were established from the relationship between observed MFY and the NASEM (2021) multivariate prediction. Two main metrics were used: (1) concordance correlation coefficient (**CCC**), as described by Lin (1989) using epiR package (Stevenson and Sergeant, 2021), and (2) the root mean square error (**RMSE**) and its bias partition, as described by Bibby and Toutenburg (1977). The CCC was split into 2 components: the bias correction factor and Pearson correlation (**r**), representing accuracy and precision, respectively. Central tendency bias, regression bias, and disturbance bias were also evaluated.

The cows were all Holstein, weighing 672 kg, and producing 29.1 kg of milk with 4.26% of fat, for a daily yield of 1,208 kg of fat (Table 1). These lactation performances are similar to those previously reported for Holstein dairy herd in the province of Québec (Ouellet et al., 2019). A slightly greater milk fat percentage was observed, which could be explained by the fact that the collection period was mostly conducted during the winter season. Milk fat concentration is known to be decreased due in part to heat stress during summer (Ouellet et al., 2019).

**Table 1:** Descriptive statistics of diets and cows used for milk fat yield predictions

Item	Mean	SD	Minimum	Maximum	n
Diet					
ADF, % of DM	23.6	3.2	17.1	35.9	541
NDF, % of DM	38.8	4.1	32.0	54.1	541
Crude fat, % of DM	2.5	0.7	1.6	4.0	541
Digestible 16:0, % of DM^1^	0.29	0.05	0.19	0.41	—
Digestible 18:3, % of DM^1^	0.38	0.09	0.19	0.60	—
Ile, % of MP^1^	5.98	0.12	5.61	6.21	—
Met, % of MP^1^	2.21	0.04	2.13	2.30	—
Forage					
NDF, % of forage DM	51.3	7.6	36.4	66.8	58
NDF digestibility,^2^ % of NDF	53.4	5.8	42.5	67.2	58
Cow					
Parity	2.4	1.5	1	8	541
BW,^3^ kg	672	57.7	535	841	541
DIM	202	116	22	684	541
Milk yield, kg/d	29.1	9.5	5.9	63.8	541
Milk fat concentration, %	4.26	0.64	2.09	7.13	541
Milk fat yield, g/d	1,208	370	246	1,929	541
Herd average					
Lactating cows, n	44.8	16.9	23	98	23
Parity	2.4	0.28	1.8	3.0	23
BW, kg	676	20	632	705	23
DIM	184	27	129	231	23
Milk yield, kg/d	30.3	3.2	23.5	36.2	23
Milk fat concentration, %	4.20	0.20	3.89	4.62	23
Milk fat yield, g/d	1,252	127	970	1,494	23

1 Table values from NASEM (2021).

2 48-h in vitro incubation.

3 Estimated using thoracic circumference (Yan et al., 2009).

Dairy rations were based on mixed silages (99%), among which 14% contained legume silage, 19% grass silage, and 36% corn silage. Corn grain was the main energy concentrate, being present in 86% of diets, whereas soybean meal was used as a protein source in 62% of diets. Rumen-protected fat was fed to 41 animals, all in the same herd.

Dry matter intake derived from the animal factors (equation 1) averaged 24.0 kg/d, whereas intake derived from feed and animal factors (equation 2) averaged 20.9 kg/d (Table 2). From these 2 estimated intakes, MFY were predicted to be 1,164 and 1,040 g/d, respectively. These predictions resulted in CCC of 0.68 and 0.63, and in RMSE of 244.8 and 292.0 g/d (20.2 and 24.2% of observed mean), respectively. By comparison, in the original model description by Daley and Hanigan (2019), CCC was established at 0.81 and RMSE at 14.1% of observed mean. Moreover, in its evaluation, the NASEM (2021) committee later reported a CCC of 0.62 and a RMSE of 205 g/d (18.9% of observed mean).

**Table 2:** Performance of milk fat yield predictions from National Academies of Sciences and Medicine (NASEM) model using 2 equations to estimate DMI

Variable	Model basis to estimate DMI	
	Animal factors	Feed and animal factors
DMI,^1^ kg/d	24.0 (SD = 2.5)	20.9 (SD = 2.8)
Milk fat yield		
Observed mean, g/d	1,208	1,208
Predicted mean, g/d	1,164	1,040
Concordance correlation coefficient^2^	0.68	0.63
r	0.79	0.77
Bias correction factor	0.86	0.82
Root mean square error, g/d	244.8	292.0
Central tendency bias,^3^ % of MSE	3.2	32.9
Regression bias, % of MSE	9.4	0.9
Disturbance bias, % of MSE	87.4	66.2

1 Estimated based on NASEM (2001).

2 Based on Lin (1989).

3 Mean square error.

The model selected to estimate DMI had a major impact on predicted MFY (Figure 1). The calculation using intake derived from animal factors (equation 1) yielded the most precise and accurate estimate, as shown by greater CCC, and lesser RMSE and central tendency bias as compared with the intake derived from feed and animal factors (equation 2). Fat yield based on the latter equation was shown to yield a prediction with lesser r value and bias correction factor, and greater central tendency bias.

**Figure 1:**
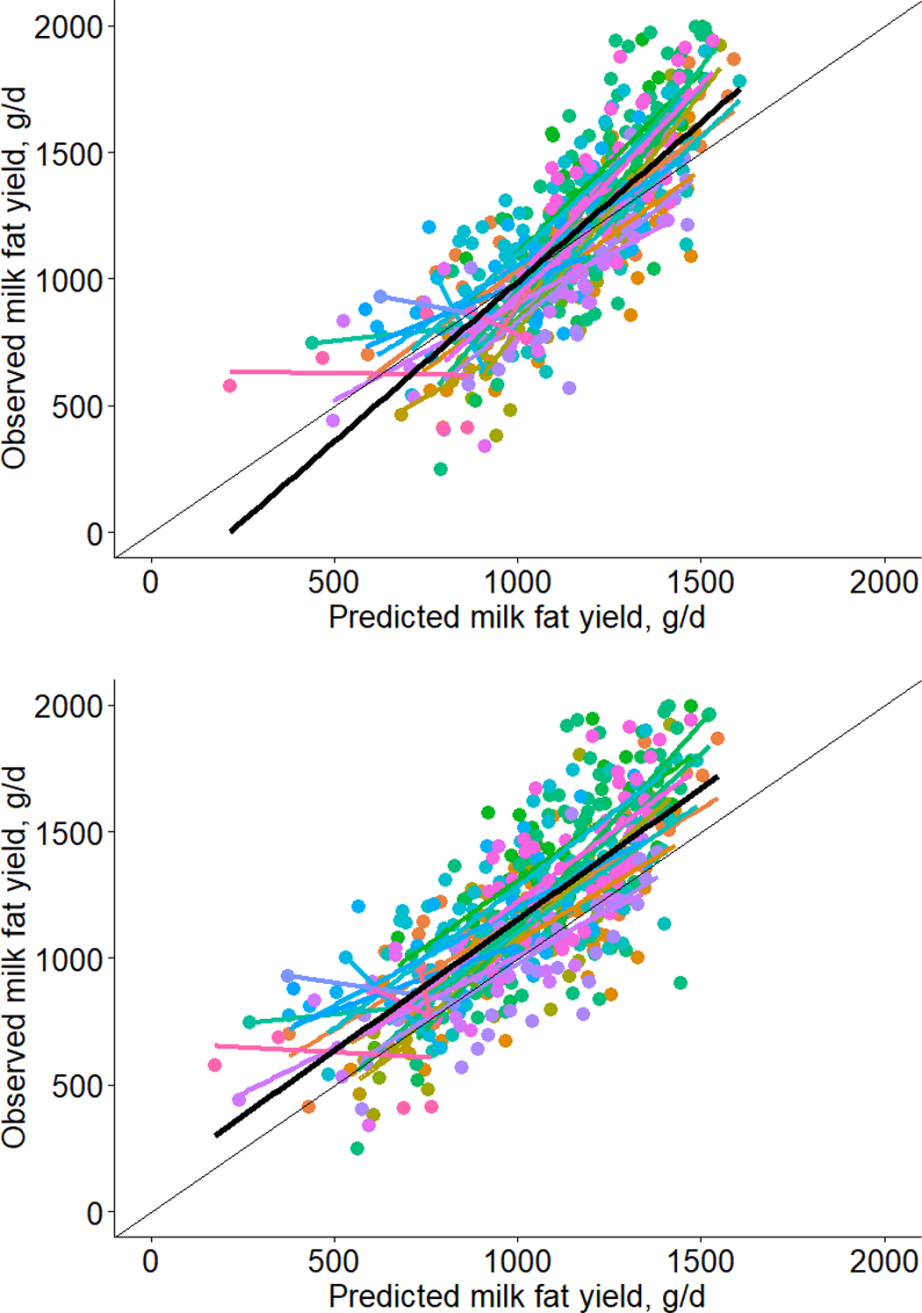
Associations between observed and predicted milk fat yield based on DMI estimated using animal factors (equation 1; upper panel) or feed and animal factors (equation 2; lower panel). Farms (n = 23) are represented by different colors. Each point corresponds to an animal. The regressions for individual farms are shown by colored lines, whereas the bold, black line represents the overall regression.

Equation 1 uses net milk energy output (*Milk_NEuse_Target_*) to predict DMI, whereas equation 2 rather includes actual milk yield (*Milk_Prod_Target_*). Yet, fat contributes to more than half of the energy of milk (NASEM, 2021), in addition to being the major component whose concentration is the most variable. This difference may have contributed to a better accuracy of equation 1 using milk energy to estimate DMI in a model to predict MFY.

The issued NASEM model has been developed and cross-validated using data from the scientific literature (Daley et al., 2022). Measurements were performed under controlled conditions, and actual DMI was available. A lower model accuracy was expected with the current external evaluation using farm data. A benchmark in this situation would be to apply an “at least as good as” criterion when comparing CCC and RMSE between the initial cross-validation as a reference (CCC = 0.62; RMSE = 18.9% of observed mean) and our external evaluation (CCC = 0.68; RMSE = 20.2% of observed mean). These points of comparison would support the utilization of the model for decision-making at the farm.

The current evaluation has been conducted with cows producing between 643 and 1,786 g of fat per day (5 to 95% CI). Further evaluation will have to include data on cows with greater MFY. A second limitation of the current evaluation is related to the fact that our database lacked information regarding cow BCS and dietary fatty acid and AA profiles. The impacts of actual measurements of these variables on the accuracy of MFY prediction remain to be evaluated.

In conclusion, the NASEM (2021) equation turned out to be a reliable tool to predict the yield of milk fat. Estimation of DMI of lactating cows was found to have a significant impact on the accuracy of the predicted values. Nevertheless, the application of this model appears as a valuable method to predict the impact of diet changes on MFY in commercial herds.
